# Mutual maintenance of PTSD and physical symptoms for Veterans returning from deployment

**DOI:** 10.1080/20008198.2019.1608717

**Published:** 2019-05-21

**Authors:** Lisa M. McAndrew, Shou-En Lu, L. Alison Phillips, Kieran Maestro, Karen S. Quigley

**Affiliations:** a War Related Illness and Injury Study Center, Veterans Affairs, New Jersey Health Care System, East Orange, NJ, USA; b Department of Educational and Counseling Psychology, University at Albany, Albany, NY, USA; c Department of Biostatistics and Epidemiology, School of Public Health, Rutgers University, New Brunswick, NJ, USA; d Psychology Department, Iowa State University, Ames, USA; e Department of Veterans Affairs, Bedford Memorial Hospital, Bedford, MA, USA; f Interdisciplinary Affective Science Laboratory, Northeastern University, Boston, USA

**Keywords:** Post-traumatic stress disorder, pain, symptom, comorbid, mutual maintenance, medically unexplained symptoms, Veteran, trastorno de estrés postraumático, dolor, síntoma, comórbido, mantención mutua, síntomas medicamente inexplicables, veterano, 创伤后应激障碍，疼痛，症状，共病，相互维持，医学上无法解释的症状，退伍军人

## Abstract

**Background**: The mutual maintenance model proposes that post-traumatic stress disorder (PTSD) symptoms and chronic physical symptoms have a bi-directional temporal relationship. Despite widespread support for this model, there are relatively few empirical tests of the model and these have primarily examined patients with a traumatic physical injury.

**Objective**: To extend the assessment of this model, we examined the temporal relationship between PTSD and physical symptoms for military personnel deployed to combat (i.e., facing the risk of death) who were not evacuated for traumatic injury.

**Methods**: The current study used a prospective, longitudinal design to understand the cross-lagged relationships between PTSD and physical symptoms before, immediately after, 3 months after, and 1 year after combat deployment.

**Results**: The cross-lagged results showed physical symptoms at every time point were consistently related to greater PTSD symptoms at the subsequent time point. PTSD symptoms were related to subsequent physical symptoms, but only at one time-point with immediate post-deployment PTSD symptoms related to physical symptoms at three months after deployment.

**Conclusion**: The findings extend prior work by providing evidence that PTSD and physical symptoms may be mutually maintaining even when there is not a severe traumatic physical injury.

## HIGHLIGHTS

• We followed soldiers from before to after combat and found a high comorbidity of PTSD and physical symptoms.• PTSD and physical symptoms were mutually maintaining among soldiers who did not experience a traumatic injury resulting in hospitalization.

## Abstract (es)

**Antecedentes**: El modelo de mantención mutua propone que los síntomas de TEPT y los síntomas físicos crónicos tienen una relación temporal bidireccional. A pesar del apoyo ampliamente difundido para este modelo, existen relativamente pocas pruebas empíricas del modelo y éstas sólo estudian a pacientes hospitalizados por una lesión traumática.

**Objetivo**: Para extender el estudio de este modelo, estudiamos la relación temporal entre síntomas de TEPT y síntomas físicos en personal militar desplegado en combate (por ej., enfrentando riesgo de muerte), quienes no fueron evacuados por lesión traumática.

**Métodos**: El presente estudio utilizó un diseño prospectivo de correlaciones cruzadas para comprender las relaciones entre síntomas de TEPT y síntomas físicos antes, inmediatamente después, 3 meses después, y 1 año después tras el despliegue de combate.

**Resultados**: Los resultados de correlación cruzada mostraron que los síntomas físicos en cada punto temporal estuvieron consistentemente relacionados con mayores síntomas de TEPT al siguiente punto temporal (λ1=.15, λ2=.16 and λ3 =.27). Los síntomas de TEPT se relacionaron con síntomas físicos posteriores, pero sólo en un punto temporal; los síntomas de TEPT inmediatamente tras despliegue se relacionaron con síntomas físicos a los 3 meses tras despliegue (=.28).

**Conclusión**: Los resultados extienden los trabajos previos al proveer evidencia de que síntomas de TEPT y síntomas físicos se mantienen mutuamente incluso cuando no existe una lesión física traumática severa.

## Abstract (zh)

**背景**：相互维持模型提出创伤后应激障碍（PTSD）症状和慢性身体症状具有双向时间关系。尽管对该模型得到广泛支持，但该模型的实证研究相对较少，并且仅考察了受伤住院的患者。

**目的**：为了扩展对该模型的研究，我们检查了参战军人（面临死亡风险）的PTSD与身体症状之间的时间关系，被试人员没有因受伤撤离。

**方法**：本研究使用前瞻性设计来了解战争服役之前、之后、之后3个月和之后1年的PTSD与身体症状之间的交叉滞后关系。

**结果**：交叉滞后结果显示，每个时间点的身体症状始终与随后的时间点的更多的PTSD症状相关（1 = .15, 2 = .16和3 = .27）。创伤后应激障碍的症状与随后的身体症状有关，但仅在一个时间点；刚开始服役后PTSD症状与服役后3个月的身体症状有关（= .28）。

**结论**：该研究结果扩展了以前的工作，提供了在没有严重的创伤性身体伤害的情况下，PTSD和身体症状相互维持的证据。

There is a high comorbidity between post-traumatic stress disorder (PTSD) and chronic physical symptoms (Noel et al., 2016). An estimated 50–80% of patients with PTSD have chronic physical symptoms (Amital et al., 2006; Beckham et al., 1997; Shipherd et al., 2007) and over 20% of patients with chronic physical symptoms (e.g., pain lasting 6 months or longer) have PTSD (Siqveland, Hussain, Lindstrom, Ruud, & Hauff, 2017). A meta-analysis of cross-sectional studies found an effect size of .46 (k = 16) between PTSD and physical symptoms and an effect size of .23 (k = 26) between PTSD and pain symptoms (Gupta, 2013; Pacella, Hruska, & Delahanty, 2013).

Those with both PTSD and chronic physical symptoms report greater severity of symptoms (Morasco et al., 2013; Vaegter, Andersen, Harvold, Andersen, & Graven-Nielsen, 2017), worse prognosis (Morasco et al., 2013; Rosenbloom, Khan, McCartney, & Katz, 2013) and greater disability (Akerblom, Perrin, Rivano Fischer, & McCracken, 2017; Martin, Halket, Asmundson, Flora, & Katz, 2010; Outcalt et al., 2015) than those with only PTSD symptoms or only physical symptoms. This comorbidity also complicates treatment efforts, leading to lower engagement with treatment (Outcalt, Hoen, Yu, Franks, & Krebs, 2016) and greater opioid use (Seal et al., 2012) as compared to those with only one condition. The high prevalence and greater severity of PTSD and chronic physical symptoms when they are comorbid, suggest that rather than being distinct they are ‘intricately connected’ (Beckham et al., 1997; Sharp & Harvey, 2001) and has spurred an interest in understanding the temporal relationship between the PTSD and chronic physical symptoms (Gordon JG Asmundson, Coons, Taylor, & Katz, 2002).

Sharp and Harvey (2001), proposed that the relationship between PTSD and physical symptoms is *bidirectional* or mutually maintaining. This mutual maintenance model has become well accepted (Asmundson et al., 2002; Beck & Clapp, 2011; Brennstuhl, Tarquinio, & Montel, 2015; McLean, Clauw, Abelson, & Liberzon, 2005), despite there being relatively few longitudinal studies of the potential bidirectional relationship and a limited understanding of the *contextual* factors that impact the relevance of the model, such as the extent of the trauma and the type of physical symptoms.

The extant research finds consistent support that *baseline* levels of PTSD symptoms predict subsequent increases in physical symptoms and *baseline* levels of physical symptoms predict subsequent increases in PTSD symptoms with study baseline assessments ranging from 24 hours to 3 years after the traumatic event (Carty, O’Donnell, Evans, Kazantzis, & Creamer, 2011; Feinberg et al., 2017; Jenewein, Wittmann, Moergeli, Creutzig, & Schnyder, 2009; Lee et al., 2018; Liedl & Knaevelsrud, 2008; Ravn, Sterling, Lahav, & Andersen, 2018; Stratton et al., 2014). These same studies find inconsistent support that *increases* in PTSD (from baseline to a second time point) predict further increases in physical symptoms (from a second to a third time point) and vice versa. Three studies found that *increases* in PTSD symptoms (from baseline to the second time point) predicted increases in physical symptoms (from the second to third time point), but not vice versa (Jenewein et al., 2009; Ravn et al., 2018; Stratton et al., 2014). Two additional studies found that *increases* in physical symptoms (from baseline levels to the second time point) predicted increases in PTSD symptoms (from the second to third time point), but not vice versa (Carty et al., 2011; Lee et al., 2018). Only one study found support for a bidirectional model where increases in PTSD and physical symptoms predicted subsequent increases in physical symptoms and PTSD symptoms, respectively (Feinberg et al., 2017).

Existing research is also limited as it has primarily examined the temporal relationship of PTSD and pain for individuals who had a traumatic physical injury. There is a need for research to understand if the mutual maintenance model is relevant in other contexts. Particularly for traumatic events other than traumatic physical injury requiring hospitalization. Previous research has focused on traumatic physical injury, because a key assumption of the mutual maintenance model is that traumatic physical injury (e.g., motor vehicle crash) causes intense pain and fear, forever linking pain, the traumatic event and fear (McLean et al., 2005). Later, pain is a reminder of the physical trauma and a trigger for fear and PTSD symptoms. In this framework, the physiological (e.g., muscle tension) and psychological symptoms (e.g., avoidance) of PTSD then further increase pain.

Traumatic injury requiring hospitalization, however, is only one type of trauma. Many traumatic events (e.g., being shot at, having to kill someone) do not cause severe physical tissue damage and intense peritraumatic pain. Rather, the peritraumatic physical symptoms are comparatively milder and result from arousal (e.g., muscle tension) or less severe injuries (e.g., reverberations from shooting a gun, being knocked to the ground) (Asmundson & Katz, 2008; Blanchard et al., 2006; McAndrew, Helmer et al., 2016; McAndrew, Chandler et al., 2016; McAndrew, Teichman, Osinubi, Jasien, & Quigley, 2012). This suggests that PTSD and physical symptoms could be mutually maintaining after trauma that does not lead to severe traumatic injury. It also suggests that the mutual maintenance model may be relevant for physical symptoms other than pain (e.g., muscle tension).

The goal of the current study is to determine whether there is support for the mutual maintenance model among military personnel deployed to combat in Iraq and/or Afghanistan (2005–2009) who did not experience a traumatic physical injury requiring hospitalization. Military personnel were assessed before, immediately after, 3 months after, and 1 year after deployment. Participants who were evacuated for physical injury were excluded from the study. In this population, an estimated 15–20% experience PTSD after combat deployment (Polusny et al., 2011) and 30% experience chronic physical symptoms (McAndrew, Helmer et al., 2016), with high rates of comorbidity between the conditions. To our knowledge, this is the first test of the mutual maintenance model to have data before the incident event (e.g., deployment) and to examine physical symptoms, as opposed to only examining pain. Having pre-deployment data allows us to control for pre-deployment levels of symptoms and to examine the correlation of pre-deployment symptoms with later symptoms. We hypothesized that PTSD symptoms and physical symptoms at each time point would predict increases in the other at the next time point, in support of the mutual maintenance model.

## 1. Method

### 1.1. Participants

Participants were Army National Guard and Army Reserve enlisted soldiers, recruited as a part of the HEROES Project, a prospective study designed to longitudinally assess Army personnel deploying to Operation Iraqi/Enduring Freedom (for description see (Lisa M. McAndrew et al., 2013; Quigley et al., 2012; Yan et al., 2013)). All participants were combat soldiers. Only soldiers between the ages of 18 and 60 were eligible. Participants were excluded if they (a) had high blood pressure, (b) were on medications that produced cardiovascular or autonomic effects, (c) self-reported depression, schizophrenia, or bipolar disorder, or (d) were pregnant. These exclusion criteria were chosen because of their potential impact on physical symptoms and on physiological measures obtained in the study (the latter are not reported here).

### 1.2. Procedure

This study is an analysis of The HEROES Project (details on the HEROES project including response bias and drop out can be found in our prior published work using this sample (McAndrew, Helmer et al., 2016; McAndrew et al., 2017)). The HEROES study was a longitudinal study of military personnel before they deployed, immediately after their return, 3 months after their return, and 1 year after their return from deployment. During the deployment readiness medical processing, soldiers were approached by study personnel who emphasized the voluntary nature of participation and provided study information. All study protocols were approved by the Veterans Affairs Institutional Review Boards and Research and Development Committee.

At the start of the study, 795 soldiers were eligible; 28 were excluded from analysis because they did not deploy, were officers, or were injured or killed in action. Soldiers were asked to complete questionnaires at four time points: (a) at before deployment while at the Army installation (Time 1; n = 767), (b) immediately upon or within a few days of return from deployment at the Army installation or through the mail if they did not return to the base from which they deployed (Time 2; n = 422), (c) three-months after deployment through mail (Time 3; n = 286), and (d) one-year after deployment through mail (Time 4; n = 335). We provided appropriate referrals for soldiers with significant physical or psychological health concerns. Participants could not be compensated for their participation while on active duty (Time 1 and 2), but those not on active duty at Time 3 and 4 were reimbursed for their time and effort.

### 1.3. Measures

#### 1.3.1. Physical symptoms

The Patient Health Questionnaire-15 (PHQ-15) is a self-administered questionnaire that measures physical symptom severity (Kroenke, Spitzer, & Williams, 2002). PHQ-15 was assessed at all four time points. Participants were asked ‘during the past 7 days, how much have you been bothered by…’ for each of 15 items (e.g., stomach pain; back pain; pain in arms legs or joints; menstrual cramps; headaches; chest pain). Participants were also asked to report the extent to which they were bothered, where 0 = *not bothered at all*, 1 = *bothered a little*, 2 = *bothered a lot*. Physical symptom severity was the sum of the measures and was categorized using established cut-offs: low (<5), sub-clinical (5–9), clinical (>9) (Kroenke et al., 2002).

#### 1.3.2. Posttraumatic stress disorder symptoms

Posttraumatic Stress Disorder Checklist IV-Civilian (PCL) was used to assess for PTSD symptoms (McDonald & Calhoun, 2010), with the civilian version used because we wanted to capture all potentially traumatic events, not just military-related traumas. The PCL was assessed at the three time points after deployment, but not before deployment. Participants were asked to rate ‘in the past month, how much were you bothered by…’ each of 17 symptoms (e.g., repeated, disturbing memories, thoughts, or images of a stressful experience from the past; emotional numbing or being unable to have loving feelings for those close to you) using a scale from 1 = *not at all* to 5 = *extremely*. PTSD symptoms were categorized as sub-clinically significant at a score of 25–49 and clinically significant at a score of 50 or greater (Brady, Killeen, Brewerton, & Lucerini, 2000). 

#### 1.3.3. Negative emotionality (proxy for PTSD pre-deployment)

Because PTSD symptoms were not assessed pre-deployment and because negative emotionality was strongly correlated with PTSD symptoms in this sample at the other time points (immediately after deployment *r*= .62, *p*< .01, 3 months after deployment *r*= .64, *p*< .01, one year after deployment *r*= .73, *p*< .01), we used the Negative Emotionality Scale (Waller, Tellegen, McDonald, & Lykken, 1996) as a proxy for PTSD symptoms at the pre-deployment time. The Negative Emotionality Scale is a 30 item true false questionnaire that assesses the tendency to experience negative emotions (e.g., anxiety, aggression). Example items included: ‘I often find myself worrying; my feelings are hurt rather easily.’ Negative emotionality before deployment was highly correlated with physical symptoms and PTSD symptoms across all time points (Table 2), in support of our use of negative emotionality as a proxy for PTSD symptoms.

**Table 1.** The mean (standard deviation) and percent with clinically significant symptoms of PTSD and physical symptoms across the deployment spectrum.

	Before deployment (Time 1)	Immediately After Deployment (Time 2)	3 Months After Deployment (Time 3)	One Year After Deployment (Time 4)
PTSD ^a^	9.57 (6.0)*	30.44 (11.74)/8.4%	32.86 (13.45)/13.2%	33.63 (15.50)/15.9%
Physical Symptoms ^b^	5.25 (3.93)/15.3%	7.94 (4.86)/33.5%	7.67 (5.11)/30.1%	7.71 (5.35)/33.3%

Clinically significant defined as >50 on the Posttraumatic Checklist-IV; ^b^ Clinically significant defined as >10 on the Patient Health Questionnaire-15 ; PTSD = posttraumatic stress disorder, *Before deployment negative emotionality was used as a proxy for PTSD.

**Table 2.** Bivariate correlations between posttraumatic stress disorder symptoms and physical symptoms across the deployment spectrum.

	PTSD^a^ Time 1	Phy. Sx. Time 1	PTSD Time 2	Phy. Sx. Time 2	PTSD Time 3	Phy. Sx. Time 3	PTSD Time 4	Phy. Sx. Time 4
Phy. Sx. Time 1	.46		.35	.49	.31	.46	.30	.40
PTSD Time 2	.46	.35		.54	.70	.55	.29	.41
Phy. Sx. Time 2	.25	.49	.54		.49	.60	.60	.58
PTSD Time 3	.46	.31	.70	.49		.65	.44	.48
Phy. Sx. Time 3	.36	.46	.55	.60	.65		.69	.73
PTSD Time 4	.30	.29	.60	.44	.69	.59		.64
Phy. Sx. Time 4	.18	.40	.41	.58	.48	.73	.64	

Phy. Sx. = Physical Symptoms, PTSD = Posttraumatic Stress Disorder Symptoms, Time 1 = Before Deployment, Time 2 = Immediately After Deployment, Time 3 = 3-Months After Deployment, Time 4 = 1-Year After Deployment ^a^ Before deployment negative emotionality was used as a proxy for posttraumatic stress disorder symptoms. All correlations significant at p < .01.

**Table 3.** One Year After Deployment % with low, sub-clinical and clinically significant PTSD and physical symptoms.

	PTSD Low	PTSD Sub-Clinical	PTSD Clinical	Total
Phy Sx Low (n) % within Phy Sx % within PTSD	72 64.3% 61.5%	38 33.9% 25.0%	2 1.8% 3.9%	112 100% 35%
Phy Sx Sub-Clinical (n) % within Phy Sx % within PTSD	31 31.3% 26.5%	60 60.6% 39.5%	8 8.1% 15.7%	99 100% 30.9%
Phy Sx Clinical (n) % within Phy Sx % within PTSD	14 12.8% 12.0%	54 49.5% 35.5%	41 37.6% 80.4%	109 100% 34.1%
Total % within Phy Sx % within PTSD	117 36.6% 100%	152 47.5% 100%	51 15.0% 100%	320 100% 100%

Phy Sx = Physical Symptoms, Low = 0–5, Sub-clinical = 5–10, Clinical>15; PTSD = Posttraumatic Stress Disorder, Low = 0–24; Sub-clinical = 25–49; Clinical > 50.

#### 1.3.4. Injury

The Deployment Response and Resilience Inventory Combat Exposure Scale (DRRI-CE) asks about combat experiences, such as being shot at (Vogt et al., 2013). There is one item about being injured or wounded during combat. This item was captured immediately after deployment and was used to characterize the per cent of soldiers who reported being physically injured during their deployment.

### 1.4. Data analyses

Descriptive statistics and Pearson’s correlation coefficients for the relationships between PTSD and physical symptoms at all time points were calculated. To understand the level of comorbidity between PTSD and physical symptoms, we compared the proportion of participants with none, sub-clinical and clinical levels of both PTSD symptoms and physical symptoms at one year after deployment.

Cross-lagged analyses were conducted by fitting a series of linear regression models which allowed for multiple imputations to handle missing data (Raghunathan, Solenberger, & Van Hoewyk, 2002; Schafer, 1999). The cross-lagged analysis controlled for age and gender because these are known to be related to symptom reporting. Prior to the analyses, all variables were standardized to a mean = 0 and variance = 1 so that the results of regression analyses are reported as standardized coefficient estimates. Specifically, we fitted the statistical models for the cross-lagged analyses described below:

$$\begin{array}{rl} & Y_{t}=b_{0}+b_{t-1}Y_{t-1}+g_{t-1}Z_{t-1}+e_{Y}\\ & \;\;\;\;\;\;\;\;+effectsofcovariates\left(age,gender\right) \end{array}$$

$$\begin{array}{rl} & Z_{t}=\alpha_{0}+\alpha_{t-1}Z_{t-1}+\lambda_{t-1}Z_{t-1}+\varepsilon_{z}\\ & \;\;\;\;\;\;\;\;+effectsofcovariates\left(age,gender\right). \end{array}$$

where Y_t_ denotes the physical symptoms score (from the PHQ15) and Z_t_ denotes PTSD score (from the PCL) and t denotes time (t; 1 = pre-deployment, 2 = immediately post-deployment, 3 = three months post-deployment and 4 = one-year post-deployment). Random errors are represented by ε_Y_ and ε_Z_.

For Time = 2,3,4, we fitted a series of linear regression models to obtain the correlations of physical symptoms (Y_t_) with the previous physical symptom (Y_t-1_) and previous PTSD (Z_t-1_), controlling for age and gender. We also fitted another series of linear regression models to estimate the correlations of PTSD (Z_t_) with the previous physical symptoms (Y_t-1_) and previous PTSD (Z_t-1_). Cross-sectional correlations between concurrent physical symptom and PTSD were estimated by calculating the Pearson’s correlation coefficients (r) between the residuals from these models (Y_t_’s and Z_t_’s).

For Time 1, cross-sectional correlations between concurrent physical symptoms and our proxy for PTSD – negative emotionality (Y_1_ and Z_1_) were estimated by first conducting linear regression models predicting Y_1_ and Z_1_ controlling for age and gender. We then used the residuals from these regressions to conduct Pearson’s correlation coefficient.

To handle missing data, we used multiple imputations and created 40 imputed datasets using IVEware (Raghunathan et al., 2002). On each imputed dataset, we applied the previously described approach and combined results using the SAS MIANALYSE procedure (SASv9.4). Data were similar (data not shown) when we conducted sensitivity analysis using cross-lagged structural equation modelling in SPSS with the subset of the data with complete data. 

## 2. Results

### 2.1. Demographics

Our sample was primarily male (89.7%), Caucasian (77.2%; 9.0% African American, 12.4% Hispanic) and Army National Guard (72.2%; 26.6% Army Reserve or 1.4% Active/Other). Ninety percent reported *not* being physically injured during deployment.

### 2.2. Descriptive analyses

Before deployment, 15.3% of soldiers reported clinically significant physical symptoms (see Table 1). This rose to 33.5% immediately after deployment and remained relatively steady over the year after deployment (30.1% at three months after deployment and 33.3% at one year after deployment). We did not measure PTSD symptoms before deployment. Immediately after deployment, 8.4% of soldiers reported clinically significant PTSD symptoms and this increased to 13.2% at three months after deployment and to 15.9% one year after deployment. The bivariate correlations revealed a moderate cross-sectional correlation between PTSD and physical symptoms at all time points (Table 2).

### 2.3. Comorbidity between PTSD and physical symptoms one year after deployment

We examined the co-morbidity between low, sub-clinical, and clinical categories of PTSD symptoms and physical symptoms one year after deployment (Table 3). The data showed that most participants with clinically significant PTSD symptoms at one year after return from deployment also had clinically significant physical symptoms (80%) at this same time point. Among participants with clinically significant physical symptoms at one year after deployment, fewer had clinically significant PTSD symptoms (37.6%).

### 2.4. Cross-lagged analyses

**Figure 1.**
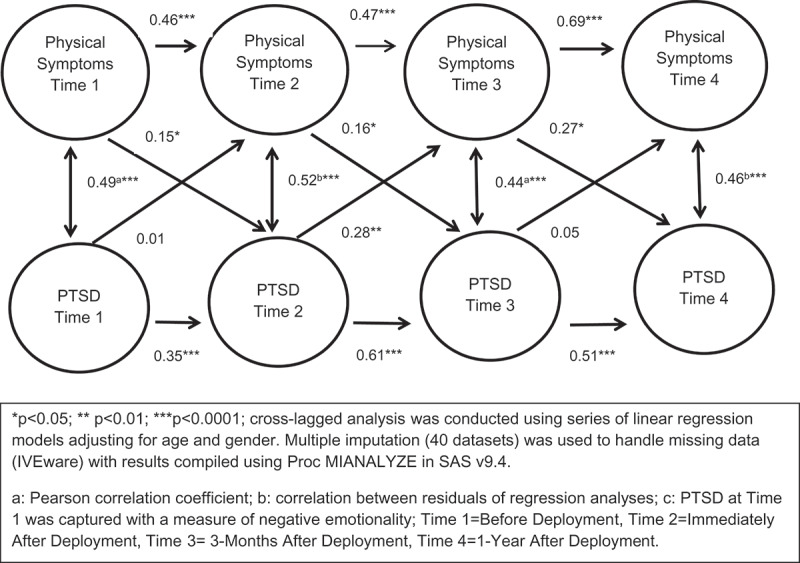
Cross-lagged relationship between PTSD and physical symptoms.

There was a moderate cross-sectional relationship between PTSD symptoms and physical symptoms (Figure 1). PTSD and physical symptoms at the same time points were moderately correlated across all time points (Before deployment: r = .49, immediate after deployment r = .52, 3 months after deployment, r = .44, and 1 year after deployment = .46; Figure 1).

The data revealed a significant longitudinal relationship between PTSD symptoms at each time point and PTSD symptoms at the subsequent time point (from before deployment to immediately after deployment λ = .35, from immediately after deployment to 3 months after deployment λ = .61 and from 3 months after deployment to one year after deployment λ = .51; see Figure 1). Similarly, there was a significant longitudinal relationship between physical symptoms from each time point to physical symptoms at the subsequent time point (before deployment to immediately after deployment β = .46, immediately after deployment to 3 months after deployment β = .47, three months after deployment to one year after deployment β = .69; Figure 1).

There was an inconsistent longitudinal relationship between PTSD symptoms at one-time point and physical symptoms at the subsequent time point. The proxy for PTSD symptoms (negative emotionality) before deployment was not related to physical symptoms immediately after deployment; PTSD symptoms immediately after deployment were related to physical symptoms 3 months after deployment (${\hat{\gamma }}_{2}$= .28), however, PTSD symptoms 3 months after deployment was not related to physical symptoms 1 year after deployment (Figure 1).

Physical symptoms at each time point were consistently related to greater PTSD symptoms at the subsequent time point. That is, physical symptoms captured before deployment were related to PTSD immediately after deployment λ_1_ = .15, physical symptoms immediately after deployment predicted PTSD symptoms 3 months after deployment λ_2_ = .16, and physical symptoms 3 months after deployment predicted PTSD one year after deployment λ_3_ = .27 (Figure 1).

## 3. Discussion

The goal of the current study was to examine the prospective temporal relationship between PTSD and physical symptoms among military personnel from before to one year after combat deployment. Overall, we found support for the mutual maintenance model of PTSD and physical symptoms; that is, PTSD and physical symptoms predicted the other at subsequent time points. Importantly, the cross-lagged analysis showed the effect was likely clinically significant; for example, increases in physical symptoms from immediately after to three months after deployment, accounted for 7% of the variance in subsequent increases in PTSD symptoms from three months to a year after deployment.

We found a bidirectional relationship of PTSD and physical symptoms from the first measurement after the event (in this case immediately after deployment) to the second time point (in this case three months after deployment). After, there was a unidirectional relationship where increases in physical symptoms predicted further increases in subsequent PTSD symptoms, but not vice versa. These findings are consistent with most previous research that showed a bidirectional relationship at first and a unidirectional relationship over time (Carty et al., 2011; Jenewein et al., 2009; Lee et al., 2018; Ravn et al., 2018; Stratton et al., 2014). The extant research is evenly split between whether the unidirectional relationship is between increases in PTSD predicting later increases in physical symptoms or increases in physical symptoms predicting later increases in PTSD symptoms. There are no apparent methodological or other reasons that appear to explain this inconsistency with at least one example of each unidirectional relationship in studies with severe trauma (Carty et al., 2011; Jenewein et al., 2009), military populations (Lee et al., 2018; Stratton et al., 2014), whether the first assessment is within a month after the trauma (Carty et al., 2011; Ravn et al., 2018) or first assessment years after the trauma (Lee et al., 2018; Stratton et al., 2014). The lack of a discernable pattern across methodologies and samples leads us to suspect that there could be a consistent bidirectional relationship that is not detected in some studies due to limitations in the methods (i.e., because symptoms are relatively stable resulting in small effect sizes that are not being detected because the assessment times are too close together and the samples insufficiently large).

Previous cross-lagged studies have often examined the development of PTSD and physical symptoms after a traumatic physical injury. The current study followed soldiers who did not have a severe traumatic physical injury but were deployed to a combat zone where they faced the possibility they could be killed. It suggests that physical symptoms that do not result from traumatic physical injury (i.e., tissue damage) can also predict increases in PTSD symptoms. Increases in physical symptoms are common during combat deployment arising from the stress of being deployed, from arousal during psychological trauma (e.g., increased heart rate while being shot at), from wear and tear of performing physically demanding jobs, and from exposure to environmental toxins and from smaller injuries that may go unreported (e.g., bruising from kneeling down to shoot a weapon). An estimated 30% of military personnel continues to experience chronic physical symptoms after deployment (McAndrew, Helmer  et al., 2016; McAndrew et al., 2012); our data suggest that the experience of these physical symptoms after deployment is associated with the initiation or increase of PTSD symptoms.

Mutual maintenance models of PTSD and physical symptoms propose that PTSD and physical symptoms become conditioned together during the trauma. While this study did not directly test this hypothesis, we did find that PTSD and physical symptoms predicted each other *and* that the cross-lagged relationship between PTSD and physical symptoms was more consistent and often stronger after deployment (i.e., during the deployment where they would be conditioned together) as compared to before deployment. Asmundson et al. (2002) suggested that in addition to being mutually maintaining, predisposing factors may cause both PTSD and physical symptoms, and our cross-lagged analysis finds support that there are individual differences before deployment that are associated with physical symptoms and PTSD symptoms after deployment. The correlation between physical symptoms before deployment to physical symptoms after development was high; and the correlation between our proxy for PTSD (negative emotionality) before deployment to PTSD symptoms after deployment was moderate. Similarly, physical symptoms before deployment, and therefore presumably before the apparent conditioning of the co-occurrence of PTSD and physical symptoms, predicts greater PTSD symptoms after deployment. These findings suggest that there are predisposing causes to both (e.g., autonomic functioning, genetic factors, personality factors, anxiety sensitivity, negative affectivity). One such factor may be premorbid trauma that previously conditioned the co-occurrence of PTSD and physical symptoms together.

A notable strength of the current study was that we had pre-deployment data. Adults are not naïve prior to traumatic events. Most adults (~60–85%) experience multiple criterion A traumatic events in their lifetime. Further, physical symptoms and negative emotionality are common in the general population and among soldiers prior to combat deployment. We found that prior to deployment, soldiers had levels of physical symptoms that were on the higher end of the general population average (~ half a standard deviation higher). Having pre-deployment data allowed us to control for pre-deployment levels of physical symptoms and negative emotionality and to examine the correlation of pre-deployment symptoms with later symptoms. A limitation was that we did not have pre-deployment levels of PTSD. The data suggests that negative emotionality is an appropriate proxy for PTSD before deployment; however, an actual measure of PTSD would have allowed us to better understand the relationship between PTSD before deployment and physical symptoms after deployment. To our knowledge, this is the first study of the mutual maintenance model to have data prior to the incident event (i.e., deployment).

Another study limitation was our measure of injury. While we trust that military personnel did not experience major traumatic injury, as those who were medically evacuated were not allowed to continue to participate in the study, our one item measure of injury may have missed minor or moderate injuries participants did not consider significant, for example, bruising. Future studies should examine differences between those who experienced no injury and those with some injuries. Finally, our study examined the directionality of the relationship between PTSD and physical symptoms at the group level. It is likely that there are individual differences in these relationships that should be explored in future studies.
